# Hyperammonemic Encephalopathy in an Adolescent Patient of Citrullinemia Type 1 With an Atypical Presentation

**DOI:** 10.7759/cureus.15109

**Published:** 2021-05-19

**Authors:** Samir Ruxmohan, Jonathan Quinonez, Jinal Choudhari, Sujan Poudel, Krunal Pandav

**Affiliations:** 1 Department of Neurology, Larkin Community Hospital, South Miami, USA; 2 Orthopedics, California Institute of Behavioral Neurosciences & Psychology, Fairfield, USA; 3 Division of Research & Academic Affairs, Larkin Community Hospital, South Miami, USA; 4 Neurology, California Institute of Behavioral Neurosciences & Psychology, Fairfield, USA

**Keywords:** type i citrullinemia, encephalomegaly, choledocholithiasis, heterozygous gene mutation, seizure

## Abstract

Citrullinemia refers to a family of autosomal recessive disorders involving the urea cycle. Three forms exist, which have different implications. Type I citrullinemia exists in both mild and severe forms. It arises due to mutations with argininosuccinate synthase leading to accumulation of ammonia and producing symptoms of lethargy, poor feeding, and seizures. Type II citrullinemia occurs due to citrin mutations involved in the urea cycle transport or during neonatal cholestasis. Management of both conditions requires low-protein diets along with arginine, sodium benzoate, and sodium phenylacetate. While traditional treatment shows improved outcomes, modifications may be necessary depending on a patient’s presentation.

We present a unique case of a 19-year-old wheelchair-bound female with a past medical history of heterozygous type I citrullinemia, seizures, and chronic encephalopathy presented to a local children’s hospital for evaluation of altered mental status with a lethargic mental state. She was initially found to have an ammonia level of 329 µmol/L and choledocholithiasis on admission. Dietary modification with intravenous dextrose and intralipids with oral lactulose saw improvement in her labs. However, her ammonia level increased to 381 µmol/L despite such interventions. Intensive care was required to normalize her serum ammonia level and clear her for a magnetic resonance cholangiopancreatography (MRCP).

We present a unique case of heterozygous type I citrullinemia with some overlap with type II citrullinemia features. Further studies are needed to understand better the observed unique presentation and long-term clinical implications associated with the disease.

## Introduction

The urea cycle (ornithine cycle) is a complex biochemical series of reactions in the mitochondria and cytosol of the liver cells and helps in the biological conversion of ammonia to urea [1]. The urea is released into the bloodstream and excreted by the kidneys. Citrullinemia is a rare autosomal recessive disorder of the urea cycle [1]. There are two subtypes of citrullinemia: type 1 and type 2. Type 1 citrullinemia (T1C) is due to the mutation of the argininosuccinate synthase (ASS) gene resulting in the deficiency or dysfunction of the enzyme ASS, which catalyzes the formation of arginosuccinate from citrulline and aspartate as a substrate in the urea cycle [1]. Classical citrullinemia leads to the state of hyperammonemia in the newborn and, if untreated, may cause neonatal death. Another variant of type 1, also called hypomorphic or mild late-onset childhood or adult-onset form, may be seen with intermittent neurological symptoms or acute crisis and may progress over the years [2,3]. Type 2 citrullinemia (T2C) is the autosomal recessive inherited disorder caused by the mutation of the *SLC25A13 *gene and usually present in adults with hyperammonemia and neuropsychiatric diseases [2,4]. The episodes of hyperammonemia are similar to those seen in the acute neonatal form, but the initial neurologic findings may be more subtle because of the older age of the patient [2,3]. It is common in East Asian descents [5].

## Case presentation

A 19-year-old wheelchair-bound, intellectually disabled Hispanic female with a past medical history of type I citrullinemia, recurrent seizures, urinary retention, and oxygen tank dependency presented to a local hospital to evaluate recurrent drowsiness.

The patient was born at full term. After birth, she experienced severe, multiple developmental delays resulting in her being wheelchair dependent. Other residual deficits include a “non-verbal” status, severe spasticity, as well as poor muscle tone. Several past electroencephalograms (EEG) demonstrated the presence of global cortical dysfunction, with a most recent video of EEG demonstrating epileptiform discharges in the left frontal head region. An MRI in 2012 revealed the presence of diffuse, severe cortical and central atrophy with ex-vacuo dilatation of the supratentorial ventricular system and prominent extra-axial cerebrospinal fluid (CSF) space. In 2015, argininosuccinate synthase 1 (ASS1) sequence analysis was done, and heterozygous c.836g>A (p.R279Q) and c.846C>a (p.y282X) mutations were found. The diagnosis of heterozygous type I citrullinemia was confirmed. Past laboratory tests done from 2016 to 2019 are mentioned in Table 1.

**Table 1:** Past laboratory test results

Laboratory test	2016	2017	2018	2019	Normal range
Citrulline	3902	3334	-	3670	15.6-46.9 µmol/L
Glutamine	1846	1606	-	821	372.8-701.4 µmol/L
Arginine	-	361	-	124	36.3-119.2 µmol/L

Before the presentation, the patient had been experiencing recurrent drowsiness for one to two months. Her family noted that she would moan in her sleep. She had also been voiding and producing bowel movements less than usual within one month. The patient and her family presented to the emergency department after attempting to awaken the patient while she was being bathed. Upon presentation to the emergency department, the patient was afebrile but mildly hypertensive. Abdominal ultrasound demonstrated the presence of cholelithiasis, choledocholithiasis, and mild hepatomegaly with fatty liver infiltration. It showed elevated ammonia levels of 280 µmol/L and 329 µmol/L (normal range: 11-32 µmol/L). The patient was admitted for further management.

On admission, intravenous fluids and intralipids were started. The patient was found to be afebrile and hemodynamically stable. Her mental state improved, but laboratory work showed increasing ammonia levels of 387 µmol/L and 322 µmol/L (normal range: 11-32 µmol/L). Other laboratory workup results are given in Table 2. She later became lethargic and was transferred to the pediatric intensive care unit (PICU). After transfer, she was lethargic but was at her baseline per family. Her diet was changed to nothing by mouth (NPO) with intravenous fluids and a fat emulsion. Arginine and sodium benzonatate were started along with an ammonia protocol diet. 

**Table 2:** Laboratory workup during hospitalization WBC, White blood cell; Hgb, hemoglobin; Hct, hematocrit; Plt, platelet; Na, sodium; K, potassium; CO_2_, carbon dioxide (serum); Cl, chlorine; Cr, creatinine; BUN, blood urea nitrogen; Mg, magnesium; Ca, calcium.

Laboratory Test	Day 1	Day 2	Day 3	Day 4	Discharge	Reference Range
WBC	5.6	6.4	5	4.7	4.1	4.5–11 x 10^7^/L
Hgb	12.5	12.2	10.1	10.3	10.9	12–15.5 g/dL
Hct	37	36.7	30.4	30.7	31.6	36-1.44-3%
Plt	179	237	206	198	152	155–450 x 10^3^/µL
Na	138	141	137	138	137	135–145 mEq/L
K	4.6	3.3	2.7	3.3	4.5	3.5–5.0 mEq/L
CO_2_	27	24	27	28	25	23–29 mEq/L
Cl	106	107	108	107	106	96–106 mEq/L
Cr	0.26	0.29	0.22	0.24	0.23	0.59–1.04 mg/dL
BUN	3	5	4	< 2	2	2.5–7.1 mmol/L
Glucose	138	84	113	91	86	65–99 mg/dL
Mg	-	-	1.5	1.8	-	1.7–2.2 mg/dL
Ca	9.7	9.5	8.9	8.8	-	8.6–10.3 mg/dL
Urine culture	-	-	-	-	Positive	Negative

Due to poor mental status, a video electroencephalogram (vEEG) was performed. The video electroencephalogram was abnormal with frequent multifocal and diffuse spikes and spike-wave discharges. The findings also show multiple potential regions for seizure onset and a diffuse encephalopathy of nonspecific etiology. The patient was given diazepam, levetiracetam, lacosamide, and lorazepam as an antiepileptic drug regimen for better control of the seizure. An MRI of the brain without contrast from a previous admission showed diffuse and severe central and cortical atrophy, with ex-vacuo dilatation of the supratentorial ventricular system and prominent extra-axial CSF spaces. Mild cerebellar atrophy was also evident. Signal intensity changes in the posterior thalami and bilateral cystic encephalomalacia involving the cerebellar hemispheres. The patient did not exhibit any seizure-like activity during the course in the PICU. No video evidence of breakthrough seizure was found on vEEG studies. She was later found to have a urinary tract infection that was treated with antibiotics. A magnetic resonance cholangiopancreatography (MRCP) revealed both cholecystolithiasis with choledocholithiasis; two stones were found in the dilated common hepatic duct. Her clinical condition improved in such a way that she was transitioned to a regular metabolic diet once ammonia levels returned to her baseline after the use of lactulose. She was discharged and transferred to another hospital to undergo an ERCP.

## Discussion

Citrullinemia is a family of autosomal recessive disorders that lead to the accumulation of ammonia and protein metabolism byproducts in the blood [1]. In summary, there are two forms of citrullinemia. T1C exists in either a heterozygous or homozygous recessive form due to mutations within the ASS gene. In contrast, T2C occurs due to mutations within the *SLC25A13 *gene; thus, citrin activity is reduced [1,5].

The homozygous form of T1C can be evident in early life. Affected infants exhibit lethargy, liver failure, loss of consciousness, and seizure leading to life-threatening conditions [2-5]. Long-term complications of T1C include recurrent hyperammonemia with growth and mental retardation, all of which require a modified diet and medications [2-5]. In contrast, heterozygous T1C is evident either in the late-childhood or early-adult years. Common symptoms include ataxia, headaches, lethargy, and scotomas [3]. Such symptoms may or may not be present depending on the disease course [3]. Newborn screening can aid in diagnosing T1C, but symptom severity and the clinical course of this disorder depend on peak ammonia levels and the age of onset of T1C [6].

T2C involves the central nervous system. Signs and symptoms include ataxia, coma, confusion, restlessness, and seizures [1,4]. They are seen either in adulthood or in the setting of infection, medication use, or pregnancy [1,4]. A variant of type II citrullinemia is seen as neonatal intrahepatic cholestasis (NIC) caused by citrin deficiency, aka neonatal-onset type II citrullinemia [7-10]. Signs and symptoms include developmental delay, dyslipidemia, fatigue, or failure to thrive [7-10]. These resolve within one year but can persist to adulthood [7-10].

The patient was diagnosed with heterozygous T1C with an ASS1 gene defect at a young age. She was found to have a milder mixed phenotype with a compound heterozygous mutation on ASS1 sequence analysis, heterozygous c.836g>A (p.R279Q) and c.846C>a (p.y282X) mutations. Since early childhood, she exhibited signs and symptoms of T1C that included developmental delay, multiple seizures, and lethargy and later became wheelchair-bound. She also experienced multiple seizure episodes that required frequent hospitalizations. An MRI without contrast back in 2012 noted brain atrophy and enlargement of the ventricles, but no specific loci for seizures were found.

Her most recent hospital admission demonstrated clinical findings and features of T2C despite a confirmed diagnosis of heterozygous T1C. A physical examination and imaging revealed choledocholithiasis, cholelithiasis, fatty liver disease, and hypertrophic cardiomyopathy (HOCM). There is an overlap of T1C and T2C symptoms, but cholelithiasis and hypertrophic cardiomyopathy are rare in T1C patients [7,10]. Fatty acid oxidation and cardiac complications have not been described in patients with citrullinemia [7]. Previous hospital visits had shown concentric and septal hypertrophy findings. She has no family history of cardiac disease or HOCM. Because of health care affordability and inaccessible hospital services, genetic testing for cardiac disease was not done. In 1980, phenylbutyrate was introduced in the United States for waste nitrogen excretion [7]. Phenylbutyrate is a strong histone deacetylase (HDAC) inhibitor, and there is a proposed connection between heart failure and HDAC inhibitors [11-13]. HDAC inhibitors act as signal responsive corepressors of the fetal cardiac gene program and cardiac growth, and so their inhibition by phenylbutyrate can be hypothesized to promote cardiac myocyte hypertrophy [7,12,14,15].

A *SCl25A13 *gene mutation results in T2C leading to citrin deficiency [4]. NIC is seen in the setting of type 2 citrullinemia [10]. Concerning citrin deficiency, three forms have been documented. The first form is NIC, seen in children with citrin deficiency having NIC; cholestasis disappears by the age of one year with proper management [10,16,17]. The second form is failure to thrive due to citrin deficiency (FTTDCD), seen in older children as failure to thrive and dyslipidemia caused by citrin deficiency [16,17]. The third form is adult-onset type II citrullinemia (CTLN2), seen in adults as recurrent hyperammonemia with neuropsychiatric symptoms in T2C in adults [16,17].

CTLN2 patients present with delirium, delusion, drowsiness, restlessness, loss of memory, flapping tremor, seizures, and coma [16,17]. These symptoms are provoked by infection, alcohol, surgery, or medications [16,17]. Our patient presented with drowsiness, urinary infection, and cholelithiasis. An MRCP showed multiple calculi in her gallbladder and the common hepatic and bile ducts dilatation, with two calculi seen in the hepatic duct. Also, fatty liver changes were noted on an abdominal ultrasound. Cholelithiasis has not been documented in the literature in a patient with citrullinemia type 1. Although there is no gene mutation causing citrin deficiency in our patient, finding cholelithiasis is present. The point of discussion here is why does the patient have cholelithiasis and cholestasis? Is it just the incidental finding unrelated to the type I citrullinemia or some genetic correlation needed to study further?

Concerning the neurological aspects of citrullinemia, episodic hyperammonemia insults a developing brain leading to cerebral edema. Over time, repeated insults lead to the development of “stroke-like” lesions that can be detected on an MRI without contrast in different brain areas, including the cingulate gyrus and insula [18]. In rare cases, encephalomalacia can also arise in the setting of T1C and T2C [2,18,19]. Encephalomalacia will predispose a patient to have a different seizure potential than the general population [2,18,19].

## Conclusions

Rare citrullinemia variants are not well studied and documented. Only a few research articles correlate with type I citrullinemia with hypertrophic cardiomyopathy, none with cholelithiasis, and some with encephalomalacia. While treating any unusual neurological patient with metabolic diseases, we may have to keep in mind the hypomorphic form of the citrullinemia type I presenting with neurological and neuropsychiatric conditions. This case puts forward the limited understanding of complications of long-term survival in cases of T1C. Hence, more research is needed to understand citrullinemia and its variants better, focusing on long-term complications.
